# Supplementary material for: Broad phylogenetic analysis of cation/proton antiporters reveals transport determinants
Source: Nat Commun. 2018 Oct 11;9:4205. doi: 10.1038/s41467-018-06770-5 (PMC6181914; doi:10.1038/s41467-018-06770-5)
Supplement: Supplementary file 1 — Supplementary Information [file 41467_2018_6770_MOESM1_ESM.pdf]

BROAD PHYLOGENETIC ANALYSIS OF CATION/PROTON ANTIPORTERS REVEALS TRANSPORT DETERMINANTS

Masrati et al.

Supplementary Information

Supplementary Figures

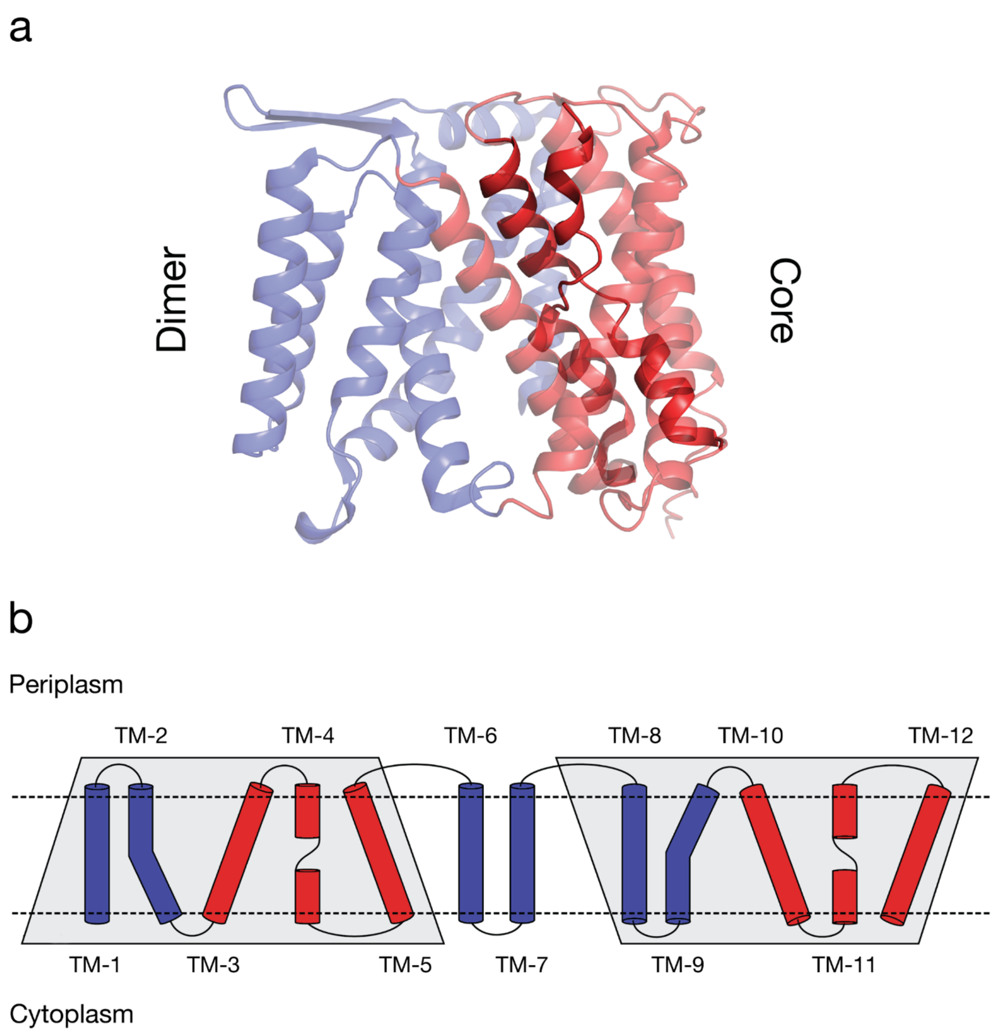

Supplementary Figure 1. The NhaA fold.

(a) Side view of EcNhaA, with its dimerization domain to the left (blue) and core domain to the right (red). The two unwound helices of the core domain that form the x-shape structure, a fingerprint of the NhaA fold, are highlighted. **(b)** Schematic two-dimensional representation of the NhaA fold. The membrane boundaries are shown as dashed lines and the helices are numbered TM-1-through-TM-12. Helices of the dimer domain are in blue, and those of the core domain are in red. The two topologically inverted repeats that characterize the NhaA fold are enclosed in gray trapezoids

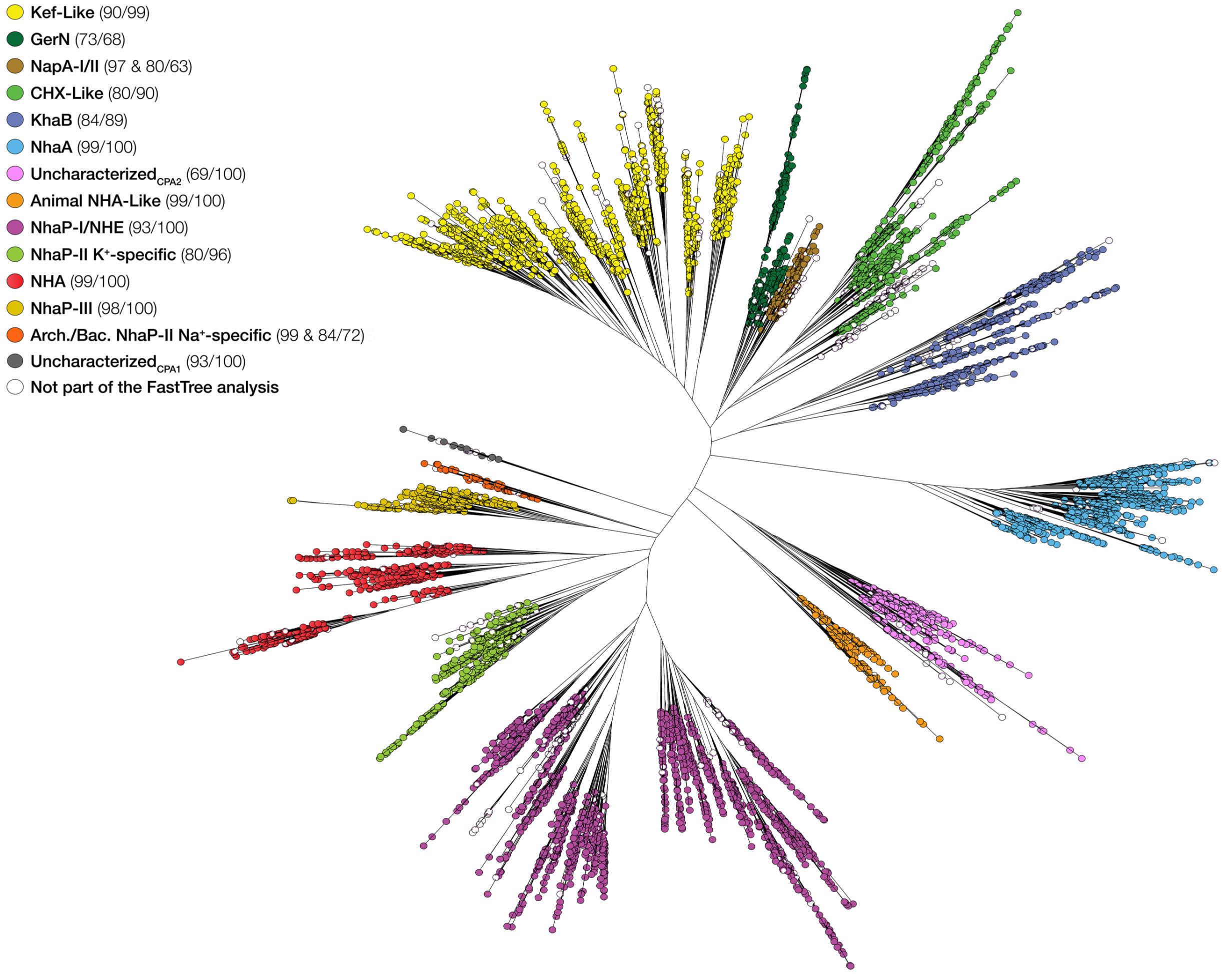

Supplementary Figure 2. IQ-TREE versus FastTree analyses.

A maximum-likelihood tree of the CPA superfamily, constructed using IQ-TREE. To compare the clade assignments in this tree with the one obtained by FastTree, we colored the different taxa in the current tree according to the FastTree's clade assignment, as specified in the above color-legend. Bootstrap values for each clade in the IQ-TREE and FastTree analyses, respectively, are given in parentheses. Overall, the two methods reproduced similar clades with the main differences being the segmentation of a few clades, such as the NapA clade, to smaller clades in the IQ-TREE analysis.

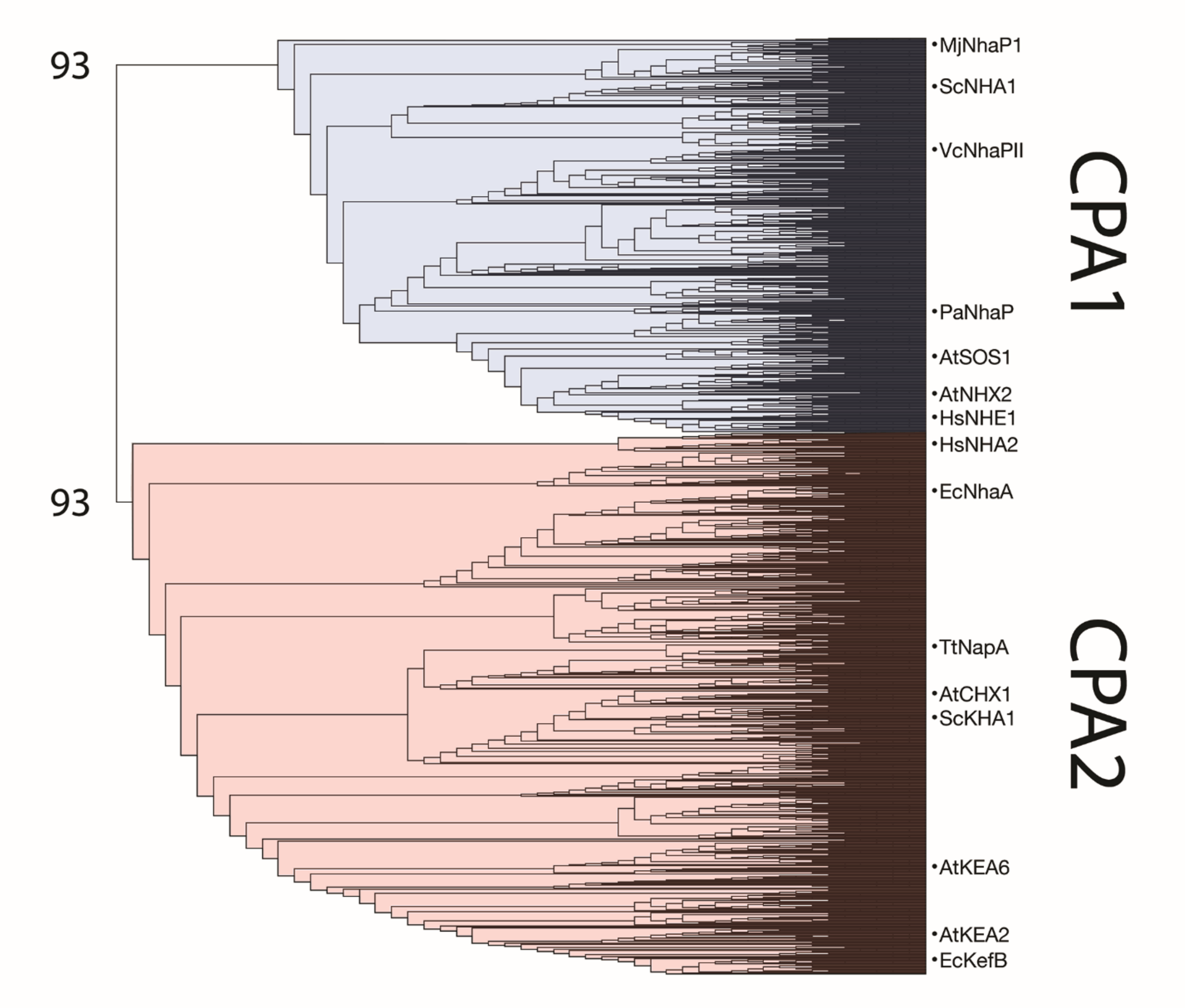

Supplementary Figure 3. CPA phylogeny.

A maximum-likelihood tree, constructed using IQ-TREE. The tree is divided into two monophyletic groups: the CPA1 (blue) and CPA2 (red) subtrees, with a bootstrap value of 93% each, based on 100 replicas. The node separating the CPA1 from the CPA2 sub-tree was chosen as the putative root of the tree. Names of representative CPAs are marked on the leaves.

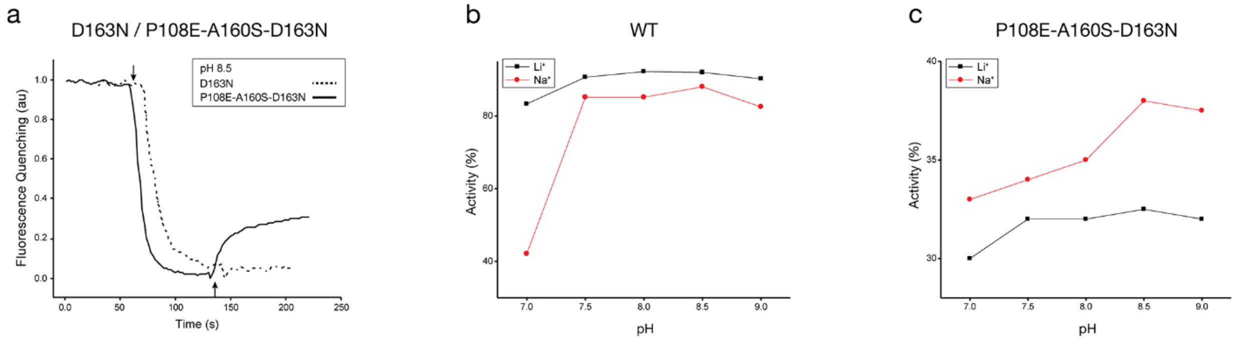

Supplementary Figure 4. The NhaA triple mutant shows Li^+/H^+ and Na^+/H^+ antiporter activity.

(a) For measuring the Li^+/H^+ antiporter activity in pH 8.5, *E. coli* EP432 cells expressing the D163N mutant (dashed line) or the P108E_A160S_D163N mutant (solid line) were grown in LBK (pH 7.0) and everted membrane vesicles were isolated. The ΔpH across the membranes was determined using acridine orange, a fluorescence probe of ΔpH. The reaction mixture (2.5 ml) contained 50–100 μg of membrane protein, 0.5 μM acridine orange, 150 mM KCl, 50 mM BTP buffer, 5 mM MgCl2 and the pH was titrated with HCl. At the onset of the reaction, D-lactate (2 mM) was added (downward facing arrow) and the fluorescence quenching was recorded until a steady-state level of ΔpH (100% quenching) was reached. Then, 10 mM LiCl/NaCl was added (upward facing arrow), and the new steady state of fluorescence obtained (dequenching) was monitored. Fluorescence dequenching indicated that protons were exiting the vesicles in response to Li^+/Na^+ influx via the antiporter in cells expressing the P108E_A160S_D163N mutant but not in cells expressing the D163N mutant. For the P108E_A160S_D163N mutant, the apparent K_m for Li^+ was 0.41±0.07 mM. All experiments were repeated at least three times with practically identical results. **(b, c)** The pH dependence of the WT molecule (**b**) and the P108E_A160S_D163N triple mutant (**c**) was determined using everted membrane vesicles as in (**a**). The maximal percent of activity (pH 8.5) is plotted versus pH.

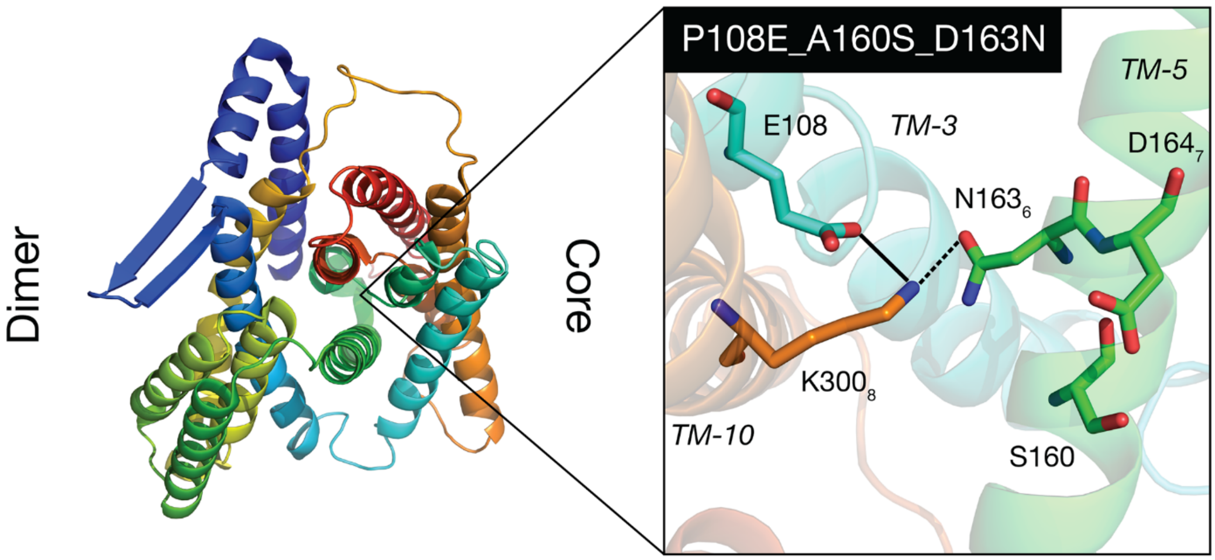

Supplementary Figure 5. Model structure of the P108E_A160S_D163N NhaA triple mutant. Left: Periplasmic view with the dimerization domain on the left and the core domain on the right. Right: Close-up view showing the mutated positions, E108, S160 and $N163_6$, and the interactions they form with other residues of the motif, as inferred from the model. E108 on TM-3 would salt-bridge with $K300_8$ on TM-10, while $K300_8$ could hydrogen bond with $N163_6$ on TM-5, preserving the conserved interaction between these two helices. As $D164_7$ was repeatedly shown to be crucial for transport, the close by S160 could potentially coordinate the substrate ion via its hydroxyl group to compensate for the loss of the carboxylate group resulting from the $D163N_6$ substitution.

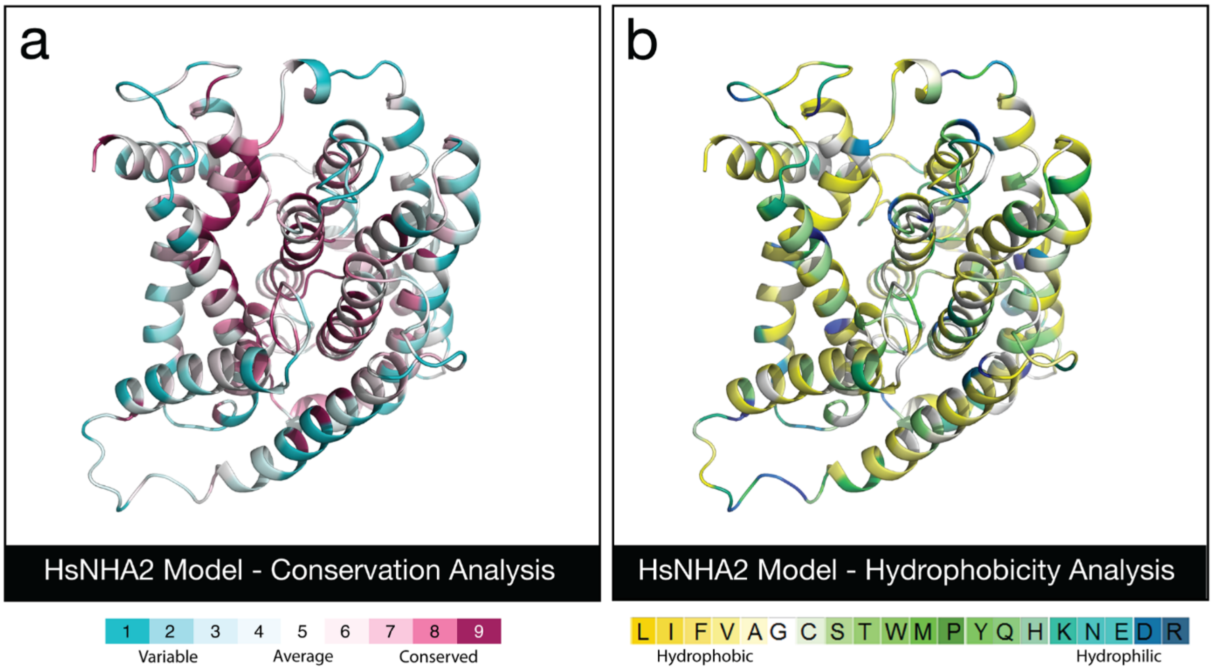

Supplementary Figure 6. HsNHA2 Homology model: *in-silico* validation.

HsNHA2 model colored according to the ConSurf evolutionary conservation color bar (**a**) and the Kessel and Ben-Tal hydrophobicity scale (**b**). As expected, the interior is polar and highly conserved and the membrane-surface is hydrophobic and variable.

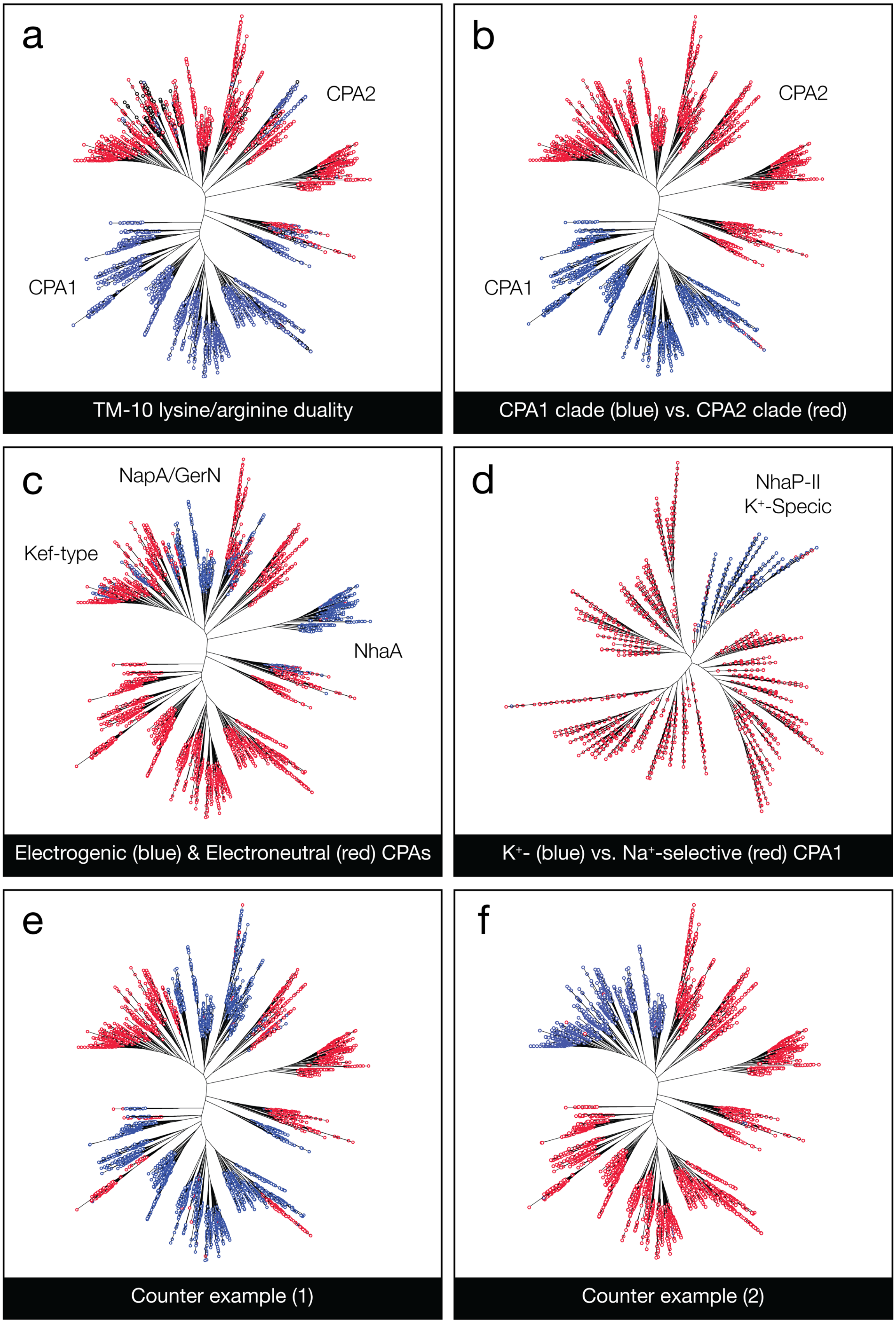
a
CPA2
CPA1
TM-10 lysine/arginine duality
b
CPA2
CPA1
CPA1 clade (blue) vs. CPA2 clade (red)
c
NapA/GerN
Kef-type
NhaA
Electrogenic (blue) & Electroneutral (red) CPAs
d
NhaP-II
K+-Specic
K+- (blue) vs. Na+-selective (red) CPA1
e
Counter example (1)
f
Counter example (2)

Supplementary Figure 7. Amino acid positions that correlate with phenotypes.

(a) Lysine/arginine duality at position 8 of the CPA motif (corresponding to position 300 in EcNhaA). Taxa in which these positions feature lysine are red, and those featuring arginine are blue. All others are black. Notably some CPA2 members feature an arginine in this position. **(b)** Positions 5 and 8 of the motif (corresponding to positions 159 and 300 in EcNhaA). Taxa in which these positions feature glutamate and arginine, respectively, are in blue. All others are red. As can be seen, this partition of the sequence pool correlates very well with the proposed segregation into the CPA1 vs. CPA2 subtrees. **(c)** Positions 6, 7 and 8 of the motif (corresponding to positions 163 and 300 in EcNhaA). Proteins that feature an acidic residue in positions 6 and 7 and a lysine in position 8 are blue, and all others are red. The two most notable groups with both acidic residue in positions 6 and 7 and a lysine in position 8 are NhaA-like and NapA/GerN-like CPAs. **(d)** Positions 1 and 4 of the motif (corresponding to positions 131 and 134 in EcNhaA) among CPA1s. Proteins that feature both serine in position 1 and alanine or serine in position 4 are in blue and all others are red. Good correlation is observed between serine and alanine in these positions and proteins belonging to the NhaP-II K^+-specific clade. **(e and f)** Two counter examples: Positions 331 (e) and 333 (f) in TtNapA, which were not included in the motif. Proteins featuring a basic residue in position 331 (e) and those featuring a glutamate at position 333 (f) are colored blue. All others are red. Though conserved, these positions does not correlate with any specific clade within the CPA superfamily, or no phenotypic trends could be attributed to them.

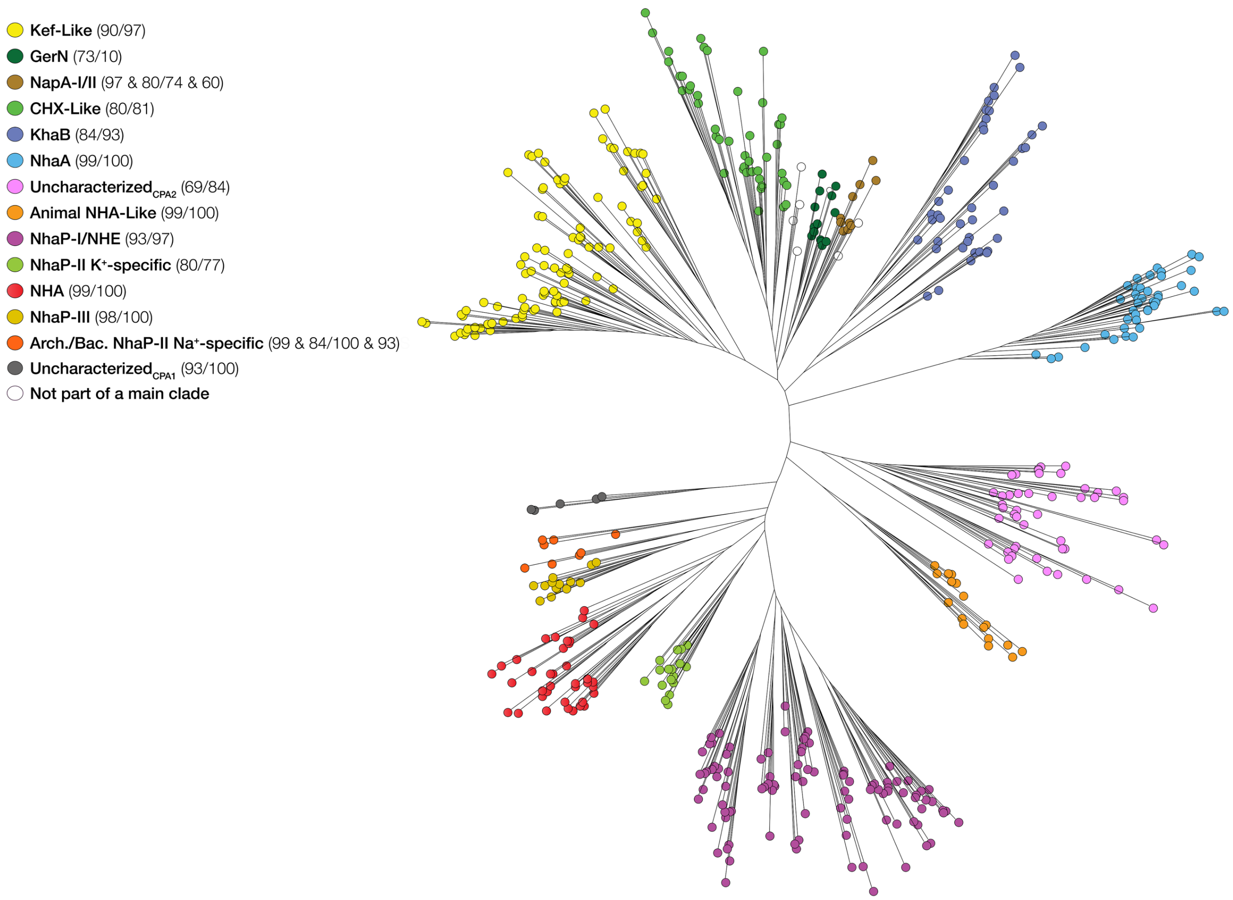

Supplementary Figure 8. IQ-TREE analysis of the 500 most divergent taxa.

A maximum-likelihood tree of the 500 most divergent taxa detected using the phylogenetic diversity analysis tool PDA and constructed using IQ-TREE. To compare the clades assignment in this tree with the one obtained from the full IQ-TREE analysis, we colored the different taxa in the current tree according to their clade assignment in the full tree, as specified in the above color-legend. Bootstrap values for each clade in the full IQ-TREE analysis and the analysis of the 500 most divergent taxa, respectively, are given in parentheses. Overall, the two analyses reproduced the same clades. One important exception is the GerN clade that presented an extremely low bootstrap value of only 10% in the analysis of the 500 most divergent taxa.

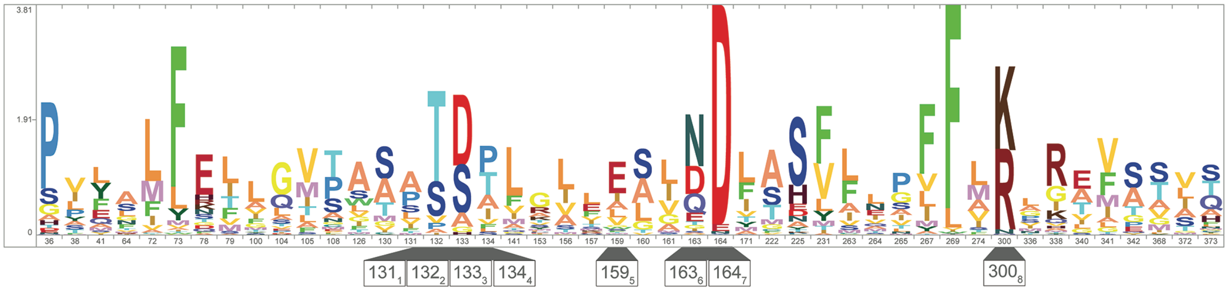

Supplementary Figure 9. Sequence logo of conserved positions among the representative CPAs.

Sequence logo showing the amino acids distribution of 46 highly conserved positions among the representative CPAs. The x-axis lists the corresponding position number in the MSA, and the y-axis represents the information content of the MSA in bits. The height of each position is proportional to its conservation and the height of each letter to its frequency in this position. The eight positions of the motif, are marked. The figure was produced using the Skylign webserver (www.skylign.org).

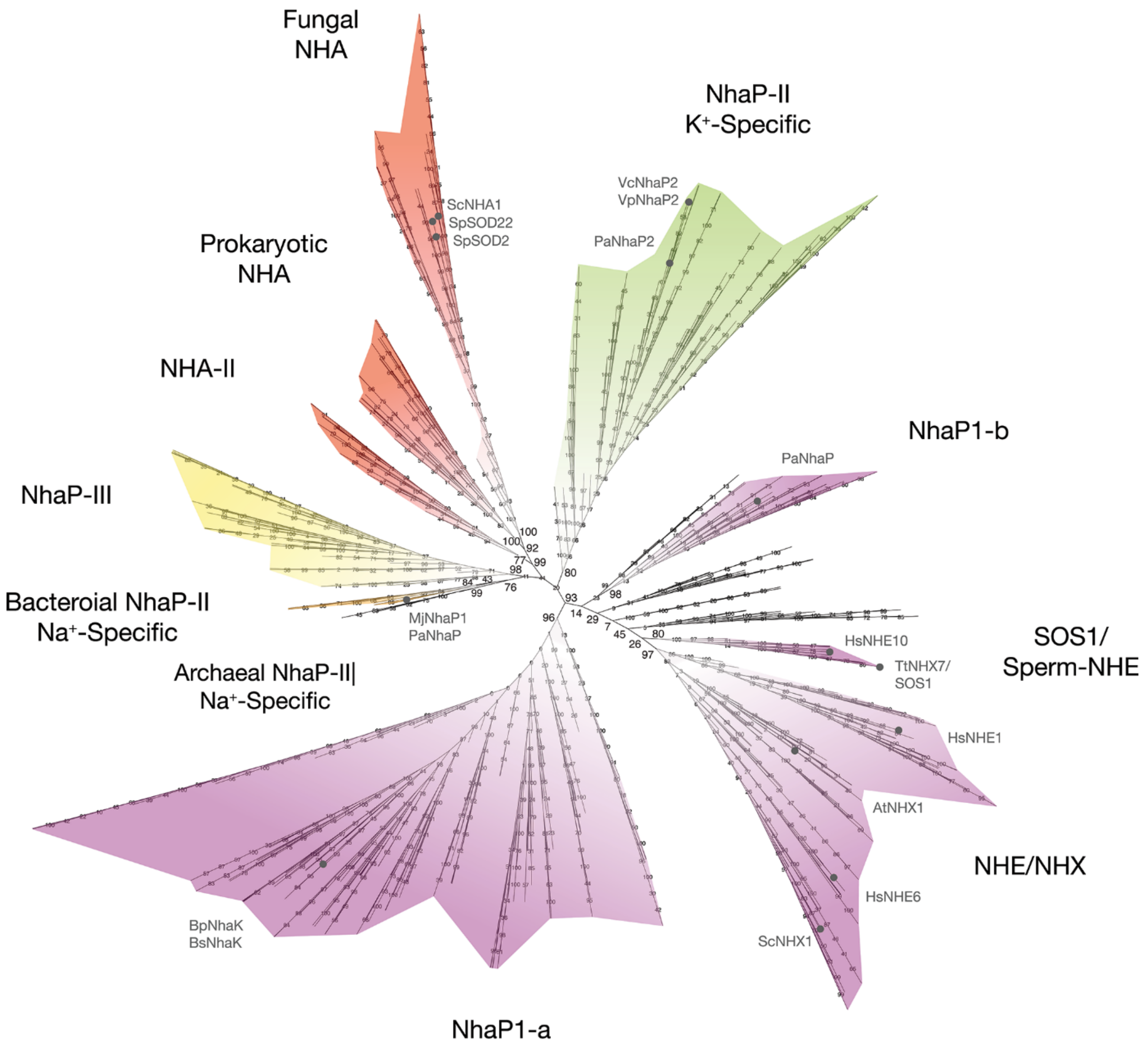

Supplementary Figure 10. The CPA1 subtree.

Unrooted tree with the main clades and sub-clades highlighted in different colors. Bootstrap values are marked for the branches that separate the main clades and sub-clades. Names of representative CPAs are presented at the leaves.

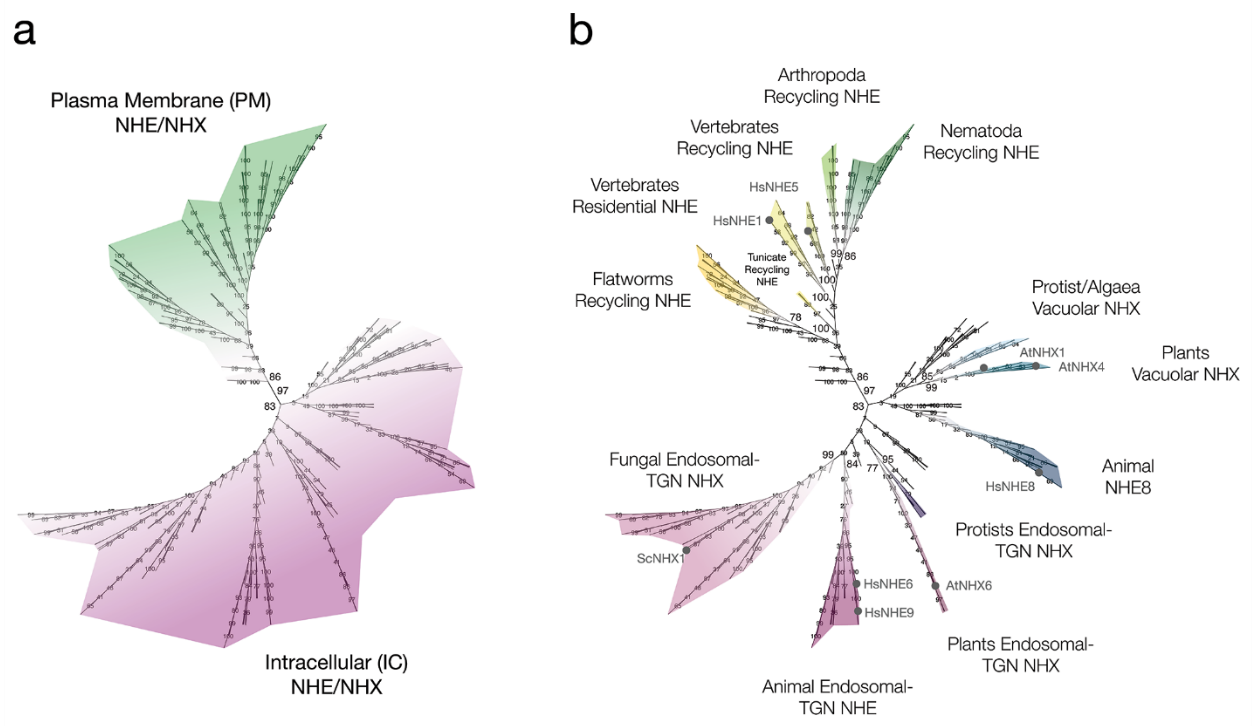

Supplementary Figure 11. The NHE/NHX subtree.

(a) Unrooted tree with the two main sub-clades comprising the NHE/NHX clade highlighted in purple (Intracellular NHE/NHX) and green (Plasma Membrane NHE/NHX). **(b)** Unrooted tree showing the inner division of the intracellular and plasma membrane NHE/NHX sub-clades. Different sub-clades are highlighted in different colors. Bootstrap values are marked for the branches that separate the main sub-clades. Names of representative CPAs are presented at the leaves.

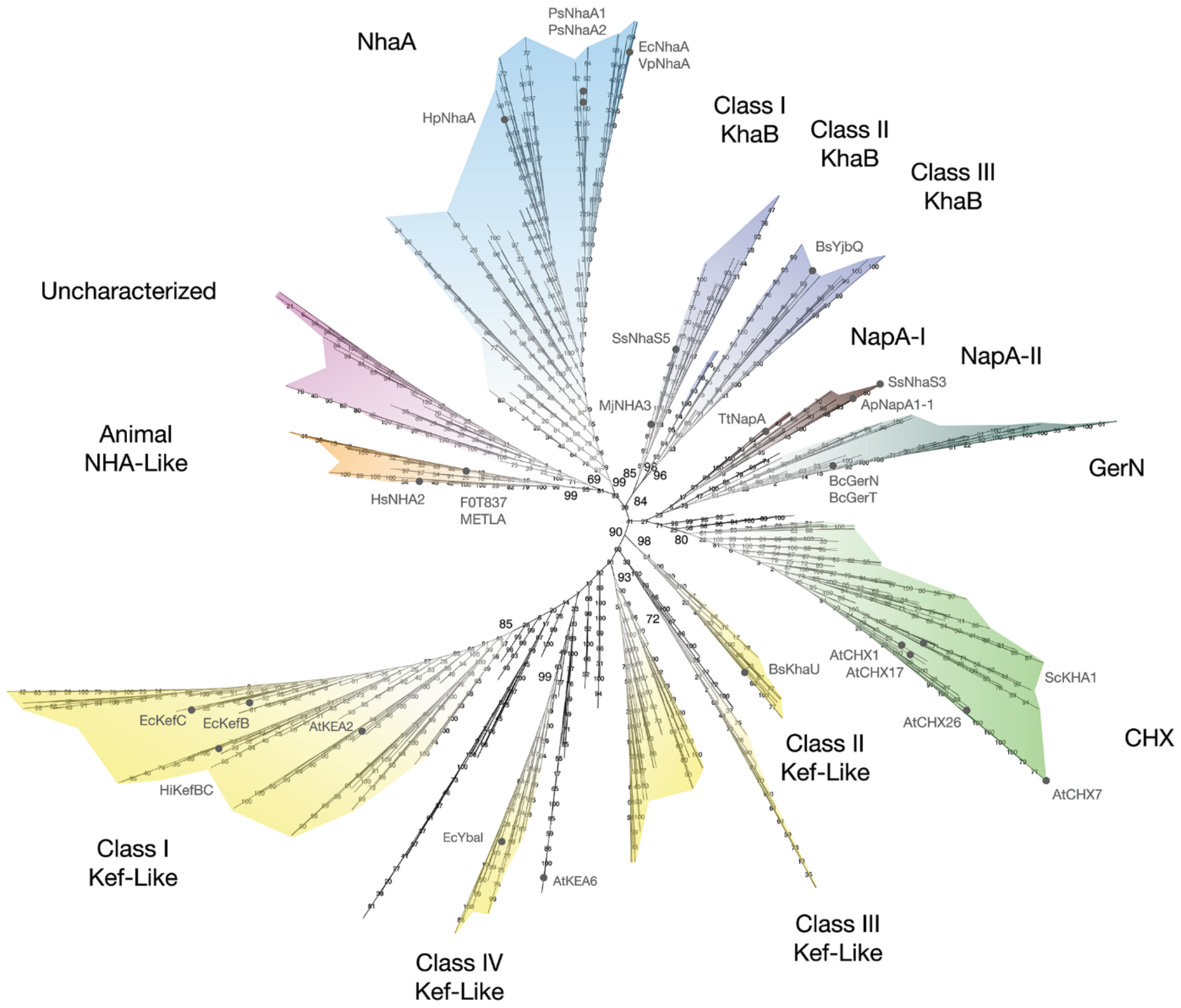

Supplementary Figure 12. The CPA2 subtree.

Unrooted tree with the main clades and sub-clades highlighted in different colors. Bootstrap values are marked for the branches that separate the main clades and sub-clades. Names of representative CPAs are presented at the leaves.

Supplementary Table 1. Primers that were used to get the mutant D163N-P108E-A160S.

Mutation	Primer Sequence	Template	Codon Change
P108E	GGTGGGATGATTGTGGAGGCATTACTCTATCTGGC	pAXH3 (WT)	CCG to GAG
D163N	GCTATTATAAACGATCTTGGG	pAXH3 (WT)	GAC to AAC
A160S	CTTTTTGATGGCTCTGAGTATTATAAACGATCTTG	pAXH3-D163N	GCT to AGT

P108E-A160S-D163N was obtained by cut and paste of the plasmids pAXH3-D160S-D163N and pAXH3-P108E by the enzymes bgl-II and mlu-I.

Supplementary Notes

Phylogenetic Tree Analysis

Both the FastTree and IQ-TREE analyses divided the phylogenetic tree into two similar groups with high bootstrap values (99% and 93% respectively). Based on the distribution of some well-characterized CPA genes, we concluded that these two subtrees correspond to the CPA1 vs. CPA2 division. It is important to note that the low sequence similarity between distant CPA members introduces a large number of gaps in the alignment used for the tree reconstruction. Therefore, positions that are important for reproducing the signal of the most probable tree are expected to be washed out by the bootstrap random sampling. This, in turn, may result in low support for some of the nodes, as we observed. We described only monophyletic groups that were assigned high bootstrap values, giving us confidence in their significance.

CPA1 subtree

The CPA1 subtree could be divided into six main monophyletic clades (Fig. 2). The largest of these, comprising 1,596 taxa, was assigned a bootstrap value of 93% and was named the NhaP-I/NHE clade. It includes bacterial NhaP type transporters alongside many of the eukaryotic CPA1 members, in particular mammalian NHEs and plants NHX transporters. This clade can be further divided into several sub-clades. Among these is one prominent eukaryotic group we named the NHE/NHX clade (bootstrap 97%, Supplementary Fig. 10), that includes representatives from protists through fungi to plants and mammals. Among these are some of the most characterized and well-studied CPA1 members, such as the human NHE1, NHE6 and NHE9 transporters (HsNHE1; HsNHE6; HsNHE9), as well as NHX1 from yeast (ScNHX1) and from *Arabidopsis thaliana* (AtNHX1). Furthermore, our analysis closely reproduced the inner division of this sub-clade, as presented in the work of Brett et al[1]. As such, the NHE/NHX clade

could be further divided into two subgroups based on subcellular localization, the plasma membrane (PM) NHE/NHX group (bootstrap 86%) and the larger Intracellular (IC) NHE/NHX group (bootstrap 83%, Supplementary Fig. 11a). Each of these two subgroups can be further divided into smaller groups that are similar, but not entirely identical, to the ones defined by Brett and colleagues[1]. The IC clade includes animal endosomal and trans-Golgi network NHEs (TGN-NHEs, bootstrap 84%), alongside protists, fungal and plants endosomal-TGN NHXs (bootstrap 95%, 99% and 77%, respectively). It also includes vacuolar NHXs from protists/algae and plants (bootstrap 85% and 99%, respectively), and animal NHE8-like transporters (bootstrap 87%, Supplementary Fig. 11b). The PM clade includes only animal genes and its inner division is, for the most part, phylum or sub-phylum based. The well-defined clades include residential-PM NHEs of vertebrates (bootstrap 100%), as well as the recycling-PM NHEs of vertebrates, arthropoda, tunicate, flatworms and nematoda (bootstrap 100%, 99%, 100% and 78%, 86% respectively, Supplementary Fig. 11b). Interestingly, vertebrates' sperm-NHEs, including the human NHE10 and NHE11, are clustered to a different clade outside of the NHE/NHX clade (Supplementary Fig. 10). This clade, which we designated the SOS1/Sperm-NHE clade (bootstrap 80%), is comprised mainly of plants and protists genes and a small number of bacterial ones. Among these is the well-studied SOS1 (NHX7) transporter from *Arabidopsis thaliana* (AtNHX7/SOS1). In the work of Brett and colleagues[1], sperm-NHEs are clustered to a third CPA clade alongside the CPA1 and CPA2 clades. This clade, named the Na^+-transporting carboxylic acid decarboxylase group (NaTDC), includes also bacterial MadA-like transporters. However, while sperm-NHEs are predicted to share the NhaA fold with other CPA1 and CPA2 members, based on the homology detection algorithm HHpred, MadA transporters do not. Thus, based on our analysis, the assignment of sperm-NHEs to the CPA1 clade is more appropriate.

Alongside these two mainly eukaryotic groups, the NhaP-I/NHE clade also includes two main prokaryotic sub-clades (Supplementary Fig. 10). The larger of the two, which we named

the Nhap-Ia group (bootstrap 96%), includes NhaP-like transporters, such as NhaK from *Bacillus pumilus* (BpNhaK) and from *Bacillus subtilis* (BsNhaK). The smaller clade, which we named the NhaP-Ib clade (bootstrap 98%), consists mostly of bacterial orthologues of *Pseudomonas aeruginosa* NhaP (PaNhaP). It also includes a very small number of eukaryotic transporters from protists. Given the small number of transporters that have been characterized experimentally, it is difficult to decisively determine what distinguishes these two clades from one another. For transporters such as BpNhaK and BsNhaK, that are part of the larger NhaP-Ia clade, lithium was shown to be a better substrate compared to sodium with lower apparent K_m values in optimal pH[2]. In contrast, lithium appears to be a poor substrate for PaNhaP, that shows homology to the smaller NhaP-Ib clade[3]. It is not clear, however, if this difference in substrate affinities applies for all members of these two clades, and it is not clear if it has any relevance to their biological functions *in vivo*. Regardless, these two groups share the same clade with many of the mammalian transporters and may serve as better model systems for understanding human CPAs. The NhaP-Ib group is particularly interesting in this respect. PaNhaP, for example, presents a relatively high 27% sequence identity to the human NHE1 exchanger. For comparison, current model systems, such as EcNhaA and MjNhaP1, show only up to 20% sequence identity to human transporters.

Alongside the NhaPI/NHE clade, we identified another monophyletic CPA1 group, and named it the NHA clade (bootstrap 99%). It includes both prokaryotic and eukaryotic taxa, among them is NHA1 from *Saccharomyces cerevisiae* (ScNHA1), as well as SOD22 and SOD2 from *Schizosaccharomyces pombe* (SpSOD22 and SpSOD2). This clade could be divided into one eukaryotic and two prokaryotic subgroups (Supplementary Fig. 10). The eukaryotic group is composed of fungal NHA protein, and was thus named the Fungal-NHA clade (bootstrap 100%). It shares a common ancestor with archaeal and bacterial taxa that form another well-defined group, which we called the Prokaryotic-NHA clade (bootstrap 100%). In Brett et al.[1], fungal NHAs were assigned to the CPA2 rather than CPA1 clade. However, as discussed in the

main text, based on the new motif identified here and the fact that NHA transporters share the unique CPA1 sequential characteristics, the assignment of fungal NHAs to the CPA1 clade seems preferable. The third group comprising the NHA clade is composed of taxa that show homology to NhaPII-K^+-specific transporters. We thus named this group the NHA-II clade (bootstrap 77%). With no experimental data, however, it is difficult to determine if there are any apparent functional differences between this and the other two NHA clades.

The third major CPA1 clade was designated the NhaPII-K^+-specific clade (bootstrap 80%). It includes 407 representatives, solely from prokaryotes. Among these are orthologues of NhaP2 from *Pseudomonas aeruginosa* (PaNhaP2), *Vibrio parahaemolyticus* (VpNhaP2) and *Vibrio cholera* (VcNhaP2). While most CPA1s bind sodium better than potassium, members of this clade, VpNhaP2 and VcNhaP2 in particular, were shown to be specific for K^+ ions[4,5]. The inner division of this clade appears to follow the taxonomic division.

The remaining three CPA1 clades are exclusively composed of archaeal and bacterial taxa. The two smaller clades, which we named the Archaeal/Bacterial NhaP-II Na^+-specific clades (bootstrap 99% and 85% respectively), include, among others, MjNhaP1and PaNhaP, both $Na^+(Li^+)/H^+$ antiporters with a solved structure[6-9]. Other taxa of these small clades show similarity to NhaP-II K^+-specific transporters. The third and larger clade, comprising 237 taxa, was named the NhaP-III clade (bootstrap 98%). Its members have some unique sequential characteristics that set them apart from other CPA1s. The majority of the representatives in this clade have a $V_1T_2G_3P_4$ … E_5 - - - I_6D_7 … R_8 motif, or a slight variation of it, instead of the more common CPA1 motif of $P_1T_2D_3P_4$ … E_5 - - - N_6D_7 … R_8. This clade does not include any well studied transporters, and its exact characteristics have yet to be discovered.

CPA2 subtree

The CPA2 subtree is mainly composed of prokaryotic taxa. The few eukaryotes are mostly fungal and plant CPA2s, with only a minor representation of higher animal taxa. The CPA2

clade divides into nine distinct monophyletic clades (Fig. 3). The most homogeneous of these in terms of the motif is the NhaA clade (bootstrap 99%), comprising 712 representatives. It is solely composed of NhaA of bacterial origin, among these, the most studied member is EcNhaA. Other members of this clade include NhaA from *Helicobacter pylori* (HpNhaA), NhaA1 and NhaA2 from *Pseudomonas syringae* (PsNhaA1/2), and NhaA from *Vibrio parahaemolyticus* (VpNhaA). Specifically, EcNhaA was shown to mediate electrogenic transport with a stoichiometry of two protons per sodium ion[10]. Given the high sequence similarity between members of this clade, all NhaA transporters are expected to be electrogenic. Low bootstrap values in the lower hierarchy of this clade, however, make it difficult to determine its inner division. The “Transporter Classification Database” classifies NhaA-like transporters to a sub-family (family 2.A.33) separate from the CPA1 and CPA2 sub-families.[11] Indeed, NhaA genes might be considered unique among other CPAs in term of their sequence and possibly slightly different architecture. However, our analysis shows that the NhaA clade is clustered as part of the CPA2 sub-tree that in turn present a high bootstrap value of 93%. Moreover, NhaA genes appear to share the conserved CPA motif that as demonstrated here, can explain their electrogenic transport mechanism. Taken together, the assignment of the NhaA clade to the CPA2 sub-tree looks preferable.

Another CPA2 member that was shown to mediate a similar electrogenic transport is TtNapA[12]. This CPA2 member is part of a smaller clade of prokaryotic genes we designated the NapA-I clade (bootstrap 94%). A slightly larger clade that includes highly similar sequences to TtNapA, such as NhaS3 from *Synechocystis sp.* (SsNhaS3) and NapA1-1 from *Aphanothece halophytica* (ApNapA1-1), was designated the NapA-II clade (bootstrap 80%). While separated in the IQ-TREE analysis, these two groups formed a unified clade in the FastTree analysis, though the bootstrap value was relatively low (bootstrap 63%). TtNapA, SsNhaS3 and ApNapA1-1 were all shown to function as $Na^+(Li^+)/H^+$ antiporters with lower apparent K_m values for lithium, as compared to sodium. Particularly, TtNapA was also shown to mediate an

electrogenic transport, similar to EcNhaA[12-14]. Based on the sequence similarity between members of these clades, it is likely that all other transporters comprising them are also electrogenic.

Another prokaryotic clade that presents high similarity to NapA-like transporters is the Spore-Germination (GerN) clade (bootstrap 73%). These CPA2s are mainly expressed in bacteria of the Firmicutes phylum, composing roughly 94% of this clade's members. Among these taxa, one can find GerN, GerT and NapA from *Bacillus cereus* (BcGerN, BcGerT and BcNapA). Many Firmicutes are known to form endospores, and BcGerN and BcGerT were shown to be required for normal spore germination and outgrowth, respectively[15-17]. This unique role may have set this group apart from other bacterial NapA-like transporters. Moreover, both BcGerN and BcGerT were shown to exchange sodium ions for protons, and BcGreN was also suggested to exchange sodium for potassium[17,18]. BcGreN appears to mediate electrogenic transport[18]. The relatively high similarity of representatives from this clade to TtNapA (~30% sequence identity) suggests that all GerN transporters are electrogenic as well. It is worth noting that analyzing the phylogenetic relationship between the 500 most divergent sequences resulted in an extremely low bootstrap value for the GerN clade (bootstrap 10%). However, some sub-clades of this branch still presented relatively high support values.

A fifth CPA2 clade, named the CHX clade (bootstrap 80%) incorporates both prokaryotic and eukaryotic taxa. The eukaryotic members of this clade include plants CHX transporters, fungal KHA transporters and a small number of protists taxa. The bacterial taxa show higher degrees of similarity to eukaryotic CHX and KHA transporters, compared to other bacterial CPAs such as NapA- and GerN-like transporters, which may suggest a common function. In this context, CHX transporters were shown to substitute mostly potassium ions for protons and are probably $K^+(Na^+)/H^+$ antiporters[19-24]. Similarly, KHA1 from *Saccharomyces cerevisiae* (ScKHA1), another member of this clade, is also a putative K^+/H^+ antiporter, although it has not been well characterized so far[25,26]. Notably, whether CHX or KHA transporters are electrogenic or not has

yet to be established. Unlike NhaA, NapA and GerN transporters, members of the CHX sub-clade lack the negatively charged residue at position 6 of the CPA motif and as a result they may indeed mediate electroneutral transport. Compared to IQ-TREE, the FastTree analysis assigned the NapA-I/II, GerN and CHX clades to one larger clade with relatively high bootstrap value (bootstrap80%).

The sixth and largest CPA2 clade is comprised of 1,467 representatives of potassium efflux (Kef) transporters. We designated it the Kef-Like clade (bootstrap 90%). It includes mostly prokaryotic transporters from both archaea and bacteria, but also a small number of eukaryotic taxa restricted to protists and plants. The activity of these unique bacterial transporters is modulated by the small molecule glutathione and some ancillary peripheral membrane proteins. Under some conditions, members of this clade are also suggested to mediate channel-like potassium efflux[27-30]. While some of the sub-groups composing the Kef-like clade either present low bootstrap values or a small number of taxa, we could still identify roughly five main monophyletic sub-clades (Supplementary Fig. 12). For convenience, we named these Class I through Class V Kef-Like sub-clades. The largest sub-clade (Class I Kef-like, bootstrap 85%) includes mostly bacterial Kef transporters with a small number of eukaryotic KEA taxa from protists and plants. Two of the most characterized bacterial potassium efflux systems are part of this sub-clade, namely KefB and KefC from *Escherichia coli* (EcKefB and EcKefC). Both EcKefB and EcKefC were shown to mediate a rapid potassium efflux that protects the cell from electrophilic reagents[31,32]. These two proteins highly resemble each other, showing 44% sequence identity, and may differ from one another in regulation paths, such as the response to glutathione metabolites[33]. The majority of taxa in this sub-clade are characterized by QD pair at positions 6 and 7 of the motif, suggesting that they mediate electroneutral transport. Indeed, measurements suggest that KEA2 from *Arabidopsis thaliana* (AtKEA2), a member of this sub-clade, is electroneutral[34]. Interestingly, two small groups (bootstrap 99% each) within this sub-clade are characterized by ED in these positions (instead of QD), making them, at least

theoretically, electrogenic. An example is KefBC from *Haemophilus influenza* (KEFX_HAEIN). Subsequently, alongside the Class I Kef-Like sub-clade we could identify three other monophyletic sub-clades that are mainly characterized by ED in these positions. We designated these the Class II, III and IV Kef-like clades (bootstrap 98%, 72% and 99%, respectively). Similar to Class I members with ED, members of these three clades could be electrogenic. All three groups are comprised of prokaryotic genes, but only the Class II clade includes a well-characterized member, the K^+/H^+ antiporter KhaU (or YhaU) from *Bacillus subtilis* (BsKhaU)[35]. Compared to EcKefC, BsKahU shows a rather narrow range of potential substrates, including potassium and rubidium ions, with K_m of 7.2 mM for potassium ions in optimal pH. On the other hand, EcKefC can exchange protons with potassium, rubidium, sodium and lithium ions, with K_m of 3.8 mM for potassium ions in optimal pH[30]. It is possible that Class-I transporters, such as EcKefC, are high-affinity-less-specific K^+ antiporters, while class II BsKhaU-like transporters are low-affinity-highly-specific K^+ exchangers. This is consistent with the role of EcKefC in responding to electrophile toxicity that requires rapid K^+ ions efflux. The Class III Kef-like sub-clade includes no well-known taxa, though it appears to be closely related to Class-II BsKhaU-like transporters. The Class IV sub-clade includes YbaI from *Escherichia coli* (EcYbaI). EcYbaI is a putative K^+/H^+ antiporter, but not much is known about its function. A fifth class of Kef-like CPA2s (bootstrap 93%) also does not include any well-characterized transporters. Similar to the majority of Class I transporters, members of this clade possess QD at positions 6 and 7 of the motif and are therefore expected to be electroneutral.

Regarding the eukaryotic members of the Kef-like clade, we could identify three main groups that include members from protists and plants only. Genes for KEA1, KEA2 and KEA3 are part of the Class I sub-clade and together with some bacterial transporters, they form a group with low support (bootstrap 40%). However, within this group, plant KEA1 and KEA2 taxa form a monophyletic group with bootstrap value of 99%, while plant KEA3 taxa, together with some protists taxa, form a second monophyletic group with bootstrap value of 92%. Apparently,

the KEA1/KEA2 group is comprised of genes localized to the chloroplast, while the KEA3 group consists of transporters localized to the thylakoid membrane[34,36]. The third eukaryotic group includes KEA4, KEA5 and KEA6-like transporters and is not part of any of the main five monophyletic sub-clades of the Kef-like clade. Instead, it is part of a sixth sub-clade with extremely low support (bootstrap 33%). Nevertheless, within this clade, these eukaryotic taxa form a monophyletic group with a bootstrap value of 86% that is distinct from KEA1-3 taxa, as observed in previous works[37,38]. Not much is known about this group, though our analysis suggests it might share a common ancestor with taxa from cyanobacteria.

The seventh clade of the CPA2 subtree includes 382 representatives from archaea and bacteria and was named the KhaB clade (bootstrap 84%). Members of this clade appear to be remotely related to NapA and GerN-like transporters. Among them are transporters such as NapA from *Methanocaldococcus jannaschii* (MjNapA, also known as MjNHA3), NhaS5 from *Synechocystis sp.* (SsNhaS5) and YjbQ from *Bacillus subtilis* (BsYjbQ). None of these is well-characterized. In general, we could identify two main types of transporters that make up this clade. The first includes two sub-clades (Class I and II, bootstrap 85% and 98%, Supplementary Fig. 12) consisting of transporters such as SsNhaS5, that comprise TD at positions 6 and 7 of the motif. Lacking the two negatively charged residues at positions 6 and 7 of the motif, these transporters are expected to be electroneutral. One experimental study suggests that ∆SsNhaS4∆SsNhaS5 mutants are not affected by high NaCl concentrations[13], though it is possible that it mediates the exchange of potassium ions with protons. This is consistent with the extra polar residue at position 4 of the motif, observed in other K^+-selective transporters and found here as well. However, it is unclear if the second type of transporters that make up this clade (Class III, bootstrap 96%, Supplementary Fig. 12) are selective for potassium as well. These taxa differ significantly from other CPAs. Nevertheless, the sensitive homology detection algorithm HHpred[39] predicts that they too share the NhaA fold. The majority of these genes present a GE pair at positions 6 and 7 of the motif, and thus lack the conserved aspartate at

position 7, a fingerprint of CPAs. They also lack the extra polar amino acid in the unwound section of TM-4 that characterizes K^+-selective CPAs and the polar or charged residue at position 6 that is involved in the conserved interaction between TM-5 and TM-10. Some of these genes feature an alternative polar residue at position 5 of the CPA motif (TM-5) that could potentially interact with the lysine/arginine on TM-10. Others have an aromatic residue at position 5 that could theoretically interact with the lysine/arginine via cation-π interactions. In cases where no polar or aromatic residue could be found at position 5, however, the phylogeny cannot account for the interaction between TM-5 and TM-10. These cases comprise only 3% of all analyzed CPAs. Assuming that they are indeed CPAs (rather than being false-positive hits of the sequence search), their differences compared to other CPAs could be indicative of different function(s), yet to be discovered.

The only mammalian CPA2 members are part of a small clade, designated the animal-NHA like clade, consisting of 133 representatives (bootstrap 99%) and featuring both prokaryotic and eukaryotic sub-clades (bootstrap 70% and 96%, respectively). Alongside the mammalian transporters, it also includes other eukaryotic taxa from arthropoda, nematode and protists. The most studied member of this clade is HsNHA2 that mediates the electroneutral exchange of sodium and lithium ions with protons[40,41]. Previously, human NHAs were proposed to be related to fungal NHA and bacterial NhaA genes[1]. However, our analysis suggests that while human NHAs are part of the CPA2 clade, fungal NHAs belong to the CPA1 clade. When it comes to bacterial NhaA taxa, although they are also part of the CPA2 clade, similar to human NHAs, the two types of transporters are clustered to different clades. Better model systems may thus include bacterial and archaeal transporters that are clustered together with mammalian NHAs, such as transporters of *Methanobacterium lacus* (F0T837_METLA) and *Clostridium aceticum* (A0A0D8IEV8_9CLOT), or their homologues.

Finally, the ninth CPA2 clade consists of 240 taxa (bootstrap 69%, named Uncharacterized) and includes prokaryotic members with no significant similarity to any well-characterized CPAs.

Supplementary Discussion

Ion selectivity

Our model for ion selectivity in CPAs calls for an extra polar residue in the binding site of K^+-selective antiporters compare to Na^+-selective ones. We could also identify a slight preference for smaller amino acids such as alanine and serine in the binding site of K^+-selective antiporters. Indeed, potassium ions are slightly larger than sodium and have a larger coordination number, often six interactions. The additional polar residue may contribute the extra coordination needed for potassium ions, and the smaller residues characterizing the ion binding site could slightly enlarge the cavity to better accommodate it (Fig. 8).

In this regard, it is important to note that in the case of transporters selectivity should result from the ability of only one ion to support the structural and dynamic changes in the protein that accompanied productive transport. This support is applied through non-covalent interactions between the ion and the transporter, which create in some stages energetically stable complexes and in other stages unstable complexes that induce conformational changes in the protein. Fully understanding the mechanism of selectivity in any given transporter require a rigorous analysis of the structural and energetic aspects of the protein-ion complex throughout the entire transport trajectory.

Since our model is based on sequence features alone, the change in the binding energy of potassium ions due to the added polar amino acid, and the resulting change in transport rate, could occur in any of the states occupied by the protein during the transport process. Hence, our model is not in odds with finding such as those presented, for example, in Alhadeff et al.[42],

which argue that both potassium and sodium bind to the protein with the same energy. The calculations of Alhadeff et al. [42] were carried out on a single state of the transporter (inward-open) and sampled conformations around this state. But the entire transport process involves other states that could potentially be responsible for the selectivity (for example, semi-occluded and occluded states). Differences in the binding energies of the different ions, in any of the other states that were not addressed, may give rise to kinetic barriers that would manifest as different transport rates.

Supplementary References

1 Brett, C. L., Donowitz, M. & Rao, R. Evolutionary origins of eukaryotic sodium/proton exchangers. *American Journal of Physiology-Cell Physiology* **288**, C223-C239 (2005).

2 Fujisawa, M., Kusumoto, A., Wada, Y., Tsuchiya, T. & Ito, M. NhaK, a novel monovalent cation/H+ antiporter of Bacillus subtilis. *Arch. Microbiol.* **183**, 411-420 (2005).

3 Kuroda, T. *et al.* A major Li+ extrusion system NhaB of Pseudomonas aeruginosa: comparison with the major Na+ extrusion system NhaP. *Microbiol. Immunol.* **48**, 243-250 (2004).

4 Radchenko, M. V. *et al.* Cloning, functional expression and primary characterization of Vibrio parahaemolyticus K+/H+ antiporter genes in Escherichia coli. *Mol. Microbiol.* **59**, 651-663 (2006).

5 Resch, C. T. *et al.* The putative Na+/H+ antiporter of Vibrio cholerae, Vc-NhaP2, mediates the specific K+/H+ exchange in vivo. *Biochemistry* **49**, 2520-2528 (2010).

6 Hellmer, J., Pätzold, R. & Zeilinger, C. Identification of a pH regulated Na+/H+ antiporter of Methanococcus jannaschii. *FEBS Lett.* **527**, 245-249 (2002).

7 Goswami, P. *et al.* Structure of the archaeal Na+/H+ antiporter NhaP1 and functional role of transmembrane helix 1. *The EMBO journal* **30**, 439-449 (2011).

8 Paulino, C., Wöhlert, D., Kapotova, E., Yildiz, Ö. & Kühlbrandt, W. Structure and transport mechanism of the sodium/proton antiporter MjNhaP1. *Elife* **3**, e03583 (2014).

9 Wöhlert, D., Kühlbrandt, W. & Yildiz, Ö. Structure and substrate ion binding in the sodium/proton antiporter PaNhaP. *Elife* **3**, e03579 (2014).

10 Taglicht, D., Padan, E. & Schuldiner, S. Proton-sodium stoichiometry of NhaA, an electrogenic antiporter from Escherichia coli. *J. Biol. Chem.* **268**, 5382-5387 (1993).

11 Saier, M. H. *et al.* The transporter classification database (TCDB): recent advances. *Nucleic Acids Res.* **44**, D372-D379 (2016).

12 Lee, C. *et al.* A two-domain elevator mechanism for sodium/proton antiport. *Nature* **501**, 573-577 (2013).

13 Inaba, M., Sakamoto, A. & Murata, N. Functional Expression in Escherichia coli of Low-Affinity and High-Affinity Na+ (Li+)/H+ Antiporters ofSynechocystis. *J. Bacteriol.* **183**, 1376-1384 (2001).

14 Wutipraditkul, N. *et al.* Halotolerant cyanobacterium Aphanothece halophytica contains NapA-type Na+/H+ antiporters with novel ion specificity that are involved in salt tolerance at alkaline pH. *Appl. Environ. Microbiol.* **71**, 4176-4184 (2005).

15 Tani, K., Watanabe, T., Matsuda, H., Nasu, M. & Kondo, M. Cloning and Sequencing of the Spore Germination Gene of Bacillus megaterium ATCC 12872: Similarities to the NaH-Antiporter Gene of Enterococcus hirae. *Microbiol. Immunol.* **40**, 99-105 (1996).

16 Thackray, P. D., Behravan, J., Southworth, T. W. & Moir, A. GerN, an antiporter homologue important in germination of Bacillus cereus endospores. *J. Bacteriol.* **183**, 476-482 (2001).

17 Senior, A. & Moir, A. The Bacillus cereus GerN and GerT protein homologs have distinct roles in spore germination and outgrowth, respectively. *J. Bacteriol.* **190**, 6148-6152 (2008).

18 Southworth, T. W., Guffanti, A. A., Moir, A. & Krulwich, T. A. GerN, an Endospore Germination Protein ofBacillus cereus, Is an Na+/H+-K+ Antiporter. *J. Bacteriol.* **183**, 5896-5903 (2001).

19 Cellier, F. *et al.* Characterization of AtCHX17, a member of the cation/H+ exchangers, CHX family, from Arabidopsis thaliana suggests a role in K+ homeostasis. *The Plant Journal* **39**, 834-846 (2004).

20 Maresova, L. & Sychrova, H. Arabidopsis thaliana CHX17 gene complements the kha1 deletion phenotypes in Saccharomyces cerevisiae. *Yeast* **23**, 1167-1171 (2006).

21 Hall, D., Evans, A., Newbury, H. & Pritchard, J. Functional analysis of CHX21: a putative sodium transporter in Arabidopsis. *J. Exp. Bot.* **57**, 1201-1210 (2006).

22 Padmanaban, S. *et al.* Participation of endomembrane cation/H+ exchanger AtCHX20 in osmoregulation of guard cells. *Plant Physiol.* **144**, 82-93 (2007).
23 Zhao, J. *et al.* AtCHX13 is a plasma membrane K+ transporter. *Plant Physiol.* **148**, 796-807 (2008).
24 Chanroj, S. *et al.* Plant-specific cation/H+ exchanger 17 and its homologs are endomembrane K+ transporters with roles in protein sorting. *Journal of Biological Chemistry* **286**, 33931-33941 (2011).
25 Ramírez, J., Ramírez, O., Saldaña, C., Coria, R. & Peña, A. A Saccharomyces cerevisiae mutant lacking a K+/H+ exchanger. *J. Bacteriol.* **180**, 5860-5865 (1998).
26 Maresova, L. & Sychrova, H. Physiological characterization of Saccharomyces cerevisiae kha1 deletion mutants. *Mol. Microbiol.* **55**, 588-600 (2005).
27 Meury, J., LEBAIL, S. & KEPES, A. Opening of potassium channels in Escherichia coli membranes by thiol reagents and recovery of potassium tightness. *The FEBS Journal* **113**, 33-38 (1980).
28 Meury, J. & Robin, A. Glutathione-gated K+ channels of Escherichia coli carry out K+ efflux controlled by the redox state of the cell. *Arch. Microbiol.* **154**, 475-482 (1990).
29 Miller, S., Ness, L., Wood, C., Fox, B. & Booth, I. Identification of an ancillary protein, YabF, required for activity of the KefC glutathione-gated potassium efflux system in Escherichia coli. *J. Bacteriol.* **182**, 6536-6540 (2000).
30 Fujisawa, M., Ito, M. & Krulwich, T. A. Three two-component transporters with channel-like properties have monovalent cation/proton antiport activity. *Proceedings of the National Academy of Sciences* **104**, 13289-13294 (2007).
31 Bakker, E. P., Booth, I., Dinnbier, U., Epstein, W. & Gajewska, A. Evidence for multiple K+ export systems in Escherichia coli. *J. Bacteriol.* **169**, 3743-3749 (1987).
32 Ferguson, G. P., Nikolaev, Y., McLaggan, D., Maclean, M. & Booth, I. R. Survival during exposure to the electrophilic reagent N-ethylmaleimide in Escherichia coli: role of KefB and KefC potassium channels. *J. Bacteriol.* **179**, 1007-1012 (1997).
33 Ferguson, G. P., McLaggan, D. & Booth, I. R. Potassium channel activation by glutathione-S-conjugates in Escherichia coli: protection against methylglyoxal is mediated by cytoplasmic acidification. *Mol. Microbiol.* **17**, 1025-1033 (1995).
34 Aranda-Sicilia, M. N. *et al.* Arabidopsis KEA2, a homolog of bacterial KefC, encodes a K+/H+ antiporter with a chloroplast transit peptide. *Biochimica et Biophysica Acta (BBA)-Biomembranes* **1818**, 2362-2371 (2012).
35 Fujisawa, M., Wada, Y. & Ito, M. Modulation of the K+ efflux activity of Bacillus subtilis YhaU by YhaT and the C-terminal region of YhaS. *FEMS Microbiol. Lett.* **231**, 211-217 (2004).
36 Kunz, H.-H. *et al.* Plastidial transporters KEA1,-2, and-3 are essential for chloroplast osmoregulation, integrity, and pH regulation in Arabidopsis. *Proceedings of the National Academy of Sciences* **111**, 7480-7485 (2014).
37 Chanroj, S. *et al.* Conserved and diversified gene families of monovalent cation/H+ antiporters from algae to flowering plants. *Frontiers in plant science* **3** (2012).
38 Rehman, H. M. *et al.* In-depth genomic and transcriptomic analysis of five K+ transporter gene families in soybean confirm their differential expression for nodulation. *Frontiers in plant science* **8** (2017).
39 Söding, J., Biegert, A. & Lupas, A. N. The HHpred interactive server for protein homology detection and structure prediction. *Nucleic Acids Res.* **33**, W244-W248 (2005).
40 Xiang, M., Feng, M., Muend, S. & Rao, R. A human Na+/H+ antiporter sharing evolutionary origins with bacterial NhaA may be a candidate gene for essential hypertension. *Proceedings of the National Academy of Sciences* **104**, 18677-18681 (2007).
41 Uzdavinys, P. *et al.* Dissecting the proton transport pathway in electrogenic Na+/H+ antiporters. *Proceedings of the National Academy of Sciences*, 201614521 (2017).

42 Alhadeff, R., Ganoth, A., Krugliak, M. & Arkin, I. T. Promiscuous binding in a selective protein: the bacterial Na+/H+ antiporter. *PloS one* **6**, e25182 (2011).
